# Implicit Selective Attention: The Role of the Mesencephalic-basal Ganglia System

**DOI:** 10.2174/1570159X21666230831163052

**Published:** 2023-08-31

**Authors:** Matteo Esposito, Sara Palermo, Ylenia Camassa Nahi, Marco Tamietto, Alessia Celeghin

**Affiliations:** 1 Department of Psychology, University of Torino, Via Verdi 10, 10124, Turin;; 2 Neuroradiology Unit, Department of Diagnostic and Technology, Fondazione IRCCS Istituto Neurologico Carlo Besta, Milan, Italy;; 3 Department of Medical and Clinical Psychology, and CoRPS - Center of Research on Psychology in Somatic Diseases, Tilburg University, PO Box 90153, 5000 LE Tilburg, The Netherlands

**Keywords:** Basal ganglia, superior colliculus, selective attention, implicit memories, blindsight, neglect, hemiplegia, anosognosia

## Abstract

The ability of the brain to recognize and orient attention to relevant stimuli appearing in the visual field is highlighted by a tuning process, which involves modulating the early visual system by both cortical and subcortical brain areas. Selective attention is coordinated not only by the output of stimulus-based saliency maps but is also influenced by top-down cognitive factors, such as internal states, goals, or previous experiences. The basal ganglia system plays a key role in implicitly modulating the underlying mechanisms of selective attention, favouring the formation and maintenance of implicit sensory-motor memories that are capable of automatically modifying the output of priority maps in sensory-motor structures of the midbrain, such as the superior colliculus. The article presents an overview of the recent literature outlining the crucial contribution of several subcortical structures to the processing of different sources of salient stimuli. In detail, we will focus on how the mesencephalic-basal ganglia closed loops contribute to implicitly addressing and modulating selective attention to prioritized stimuli. We conclude by discussing implicit behavioural responses observed in clinical populations in which awareness is compromised at some level. Implicit (emergent) awareness in clinical conditions that can be accompanied by manifest anosognosic symptomatology (*i.e*., hemiplegia) or involving abnormal conscious processing of visual information (*i.e*., unilateral spatial neglect and blindsight) represents interesting neurocognitive “test cases” for inferences about mesencephalic-basal ganglia closed-loops involvement in the formation of implicit sensory-motor memories.

## 1 INTRODUCTION

By tuning ourselves to the outside world, we may ensure that the information that matters to us the most reaches our awareness and directs our actions. The ability of the brain to recognize and highlight the most important areas of the visual field to allocate a finite amount of attentional resources is highlighted by this tuning process, which involves modulation of the visual system by both cortical and subcortical brain areas.

Selective attention is the ability to prioritize the processing of some stimuli while ignoring others. It is generally accepted that it operates by enhancing the most relevant location in space. This enhancement is first coded as a stimulus-based saliency map [1-3], which reflects a two-dimensional topographic representation of a space combining several low-level feature maps, and operates through competition, establishing the most attractive point in the space that then guides the attentional focus [4]. Selective attention is coordinated not only by the output of stimulus-based saliency maps but is also influenced by top-down cognitive factors, such as internal states, goals, or previous experiences. Together, these cognitive factors create an internal motivational saliency map that, interacting with stimulus-based saliency maps, forms a priority map that guides eye movements and/or shifts of attention [5, 6].

If perceptually salient and/or emotional stimuli can automatically capture our attention through the involvement of subcortical regions, such as the Superior Colliculus (SC), the Pulvinar (Pulv) and the Amygdala (Amg), our cognitive system can also select stimuli based on more abstract information through the mediation of frontal and parietal areas, like the frontal eye fields (FEF) and the lateral intraparietal area (LIP).

The relatively fast preattentive processing promoted by the SC-Pulv-Amg circuit is capable of detecting value-based visual stimuli (*i.e*., salience) and implementing rough responses, while FEF and LIP integrate bottom-up signals with cognitive and motivational top-down factors and then modulate the neural activity of SC and related visual cortices. In fact, the SC, FEF, and LIP are highly interconnected, with SC encoding both stimulus-based saliency maps and priority maps, while the FEF and LIP preferentially computing priority maps [6].

Past literature has mainly focused on the role of this vast cortical and subcortical network in dynamically modulating the activity in visual cortices. However, other mechanisms can also intervene in modulating selective attention, revising the stimuli values according to individual history and, as a result, revising the priority maps formed in the SC [7]. Indeed, we can implicitly learn to act and react to certain stimuli by attending to the predictability and frequency of objects and events [8] or their reward value [9]. Statistical incidental learning, namely, the extraction of regularities in the learning process not guided by a task or planned, represents one of those mechanisms.

Statistical incidental learning of the spatial distribution of the targets influences selective attention, as demonstrated by experiments on gaze patterns, which favoured regions of space where targets were more frequently presented [10]. This approach can drive feature-based as well as spatial attention [11], and it is empowered when associated with semantic [12, 13], effective, and reward [14-17] meanings.

Indeed, when spatial and temporal resources are limited, the association with emotional arousal, both positive and negative, prioritizes attention [18-21], as well as reward-associated stimuli [17]. Reward-biased attention is context-dependent, meaning that attentional capture depends on whether a stimulus feature has been previously rewarded in the same circumstances [22]. Moreover, different regions of space gain priority differently after learning, suggesting that the priority maps had been reshaped to favor sites with a history of receiving higher rewards.

The basal ganglia (BG) system plays a key part in these implicitly learnt modulation mechanisms of selective attention, including many striatal sensorimotor neurons where the stable (long-term) values of visual stimuli, learned *via* experience, are preserved [7]. These striatal neurons receive early visual input from and reproject to SC, thereby forming closed loops through which SC activity is modulated in a rapid and automatic way [23, 24].

In this article, we will focus on how subcortical areas and these striatum-collicular closed loops contribute to implicitly addressing and modulating selective attention, emphasizing how these processes can operate without explicit awareness.

## 2 FROM GAZE CONTROL TO SELECTIVE ATTENTION: THE ROLE OF THE SUPERIOR COLLICULUS

The SC is a layered structure situated on the roof (tectum) of the midbrain that is crucially involved in the orientation of the gaze and head; Fig. (**1**) shows the anatomical subdivision of SC. The SC’s layers are commonly organized into two divisions, a dorsally located visuosensory division (superficial layers) and a centrally located motor division (deep layers) [25]. The primary inputs to the superficial layers come from the retina and striate and extrastriate cortices [26], whereas the most conspicuous outputs target the posterior thalamus, primarily the lateral geniculate nucleus and the Pulv [27, 28]. The superficial visuosensory layers also project to the deep layers of SC, which instead are both multimodal and premotor. The deep layers receive inputs virtually from the entire brain and send descending efferents to brainstem nuclei and ascending outputs to anterior thalamic nuclei that supply BG and a host of cortical regions [29, 30]. Extensive literature covering multiple species highlights that SC is capable in itself of identifying biologically salient stimuli and implementing the approach and escape behaviours (*i.e*., prey capture and predator avoidance) [29, 30].

The role of SC in attention has been investigated in tasks that involve gaze control, target selection, and selective attention [31-34]. Research on build-up neurons in the SC of monkeys performing a saccadic eye movement task confirms that presaccadic activation is modulated by increasing the probability of target localization [35]. Moreover, within the deep layers of SC, there are neurons specialized in orienting the focus of attention, regardless of corresponding eye movements towards selected stimuli [36, 37], proving the crucial role of SC in selective attention. SC is also involved in the causal control of selective attention, as demonstrated by electrical microstimulation [38] or reversible inactivation of monkeys’ SC, which results in neglect-like deficits [39].

A prominent feature of SC is the presence of organized maps: superficially, a visual space map, ventrally a saccadic eye movement space map, and more recently, saliency and priority maps distributed among the different layers of the SC [32, 40]. As indicated previously, the stimulus-based saliency map computes visually conspicuous points based on low-level visual features, such as brightness, color, oriented edges and motion. All these low-level visual features are rooted in local circuits, especially in the superficial layers of the SC [32-34, 41-43]. Importantly, the superficial layers of the SC process saliency before the visual cortices, thus suggesting an ancestral mechanism that can be locally managed by this mesencephalic structure [34]. The stimulus-based saliency map output can then follow two main routes; the first one to the deep layers of SC, which in turn can implement raw responses through their direct projections to the motoneurons of the brain stem [44]; the second one to the cortical areas, where it is integrated with top-down cognitive factors, forming a priority map [6], whose output is ultimately feedbacked to the deep layers of the SC [33]. In fact, in primates, the ascending input from the superficial layers of SC through the Pulv reaches cortical areas involved in spatial attention, such as the LIP or FEF [45-47], with both areas sending direct descending projections to the deep layers of the SC. Further, the FEF is also interconnected with the dorsolateral prefrontal cortex (dlPFC), which is crucially involved in supporting deliberate shifts of attention, especially when subjects must keep in mind specific abstract rules [48, 49]. Therefore, if, on the one hand, the superficial layers of SC are capable of (autonomously) detecting potentially relevant stimuli, elaborating their value (*i.e*., their salience), and implementing fast responses [33, 34, 44], the attentional cortical network can, on the other hand, always modulate collicular activity, modifying the priority map output in the deep layers [33] (Fig. **2**).

Collicular signals can also reach the BG [23, 24]. Mounting evidence (in both humans and non-humans) highlights the role of this mesencephalic-BG network in selective attention, notably in automatically modifying the priority map output in the deep layers of the SC [7, 50], providing, moreover, new perspectives on the effect of reward on attention. Indeed, until recently, the reward was believed to influence attention indirectly by modulating task-related motivation. However, it now appears to operate directly by modifying the stimuli value (*i.e*., their salience), even when the stimulus is physically inconspicuous or irrelevant to the task [51].

## 3 THE MESENCEPHALIC-BASAL GANGLIA ARCHITECTURE

The BG system is one of the most significant components of the vertebrate brain involved in modulating neural activity of cognitive, affective and motor functions. Concerning selective attention, many studies have highlighted its crucial involvement in suppressing distractors stimuli [52-55], regulating attention-related visual changes in visual cortices [56, 57], and supporting shifts of attention [58, 59].

The BG system is characterized by *parallel closed loops* (topographically organized), each performing a different function [60]. In fact, there are three main anatomo-functional subdivisions within the BG system, determined by the specific cortical inputs to the striatum: (i) the dorsolateral and posterior putamen, and the dorsolateral rim of the body and of the tail of the caudate are sensorimotor territories; (ii) the anterior part of the putamen, most of the head of the caudate, and the middle parts of the body and of the tail of the caudate are associative territories; (iii) the ventral portions of the putamen and of the caudate are limbic territories [61, 62]. The most prominent example of the BG closed-loops configuration is the cortico-basal architecture. However, several animal studies (including primate models) have reported that the BG is also interconnected with SC, forming parallel closed loops similar to those with the cortex [23, 24]. These have the same general intrinsic organization, albeit in the first case, the thalamic nuclei transmit output signals, whereas in the second case, the input signals [23, 24] (Fig. **3**).

The mesencephalic-BG architecture includes ancient brain structures, such as the SC, and two main closed loops are identifiable within this subcortical system. The first closed-loop originates from the superficial layers of the SC (Fig. **4a**) that project to the Pulv and to the posterior lateral nucleus of the thalamus. The information then reaches the lateral territories of the body and tail of the caudate and the dorsolateral putamen, providing early visual input to the BG system. The second closed-loop (Fig. **4b**), instead, originates from the deep layers of SC that send axons to the intralaminar nuclei of the thalamus, which, in turn, project to all territories of the striatum. In both loops, neuronal information is retransmitted from the striatum to SC, mainly through the substantia nigra pars reticulata (SNpr), closing the loop [23, 24]. The SC also sends ascending projections to the substantia nigra pars compacta (SNpc), thus providing dopaminergic input to the striatum [63-65].

These two mesencephalic-BG closed loops probably process different information [23]. In fact, the superficial visuosensory layers of the SC mainly project to the sensorimotor territories of the striatum. In contrast, the deep layers, which contain multimodal and premotor neurons [25], project to all the striatal territories. Therefore, the mesencephalic-BG closed-loops that originate from the superficial layers of the SC are mainly sensorimotor, whereas those that originate from the deep layers of SC are motor, associative, limbic, and multimodal [23, 24, 50].

The two mesencephalic-BG loops just described focus on collicular inputs to the striatum. However, the deep layers of SC can also provide early signal input to the BG system through its direct projections to the subthalamic nucleus (STN) [66, 67]. The evolutionary significance of this pathway is probably to stop ongoing activity in the presence of unexpected salient stimuli [24, 50], similar to the functional role of the cortico-subthalamic pathway [68, 69]. In humans, in fact, the STN exhibits an early increase in activity after the onset of an unexpected stimulus [70, 71]. Further, studies on rodents showed that STN activation interrupts behavior, and blocking the STN blunts the interruptive effect of unexpected stimuli [72].

## 4 THE STRIATAL MODULATION OF THE SUPERIOR COLLICULUS

Practice can modulate selective attention [8], making us faster at detecting target stimuli and suppressing the interfering effect of distractors [73-75]. Interesting data collected in humans refer to a gradual reduction of attention-related activity in FEF and inferior parietal sulcus (IPS) [76], thus suggesting a transition to a less resource-dependent level of processing. Despite it having been long known, on the one hand, that SC is widely implicated in several attentional functions, such as stimuli salience processing or shifts of attention regulation [36] and, on the other hand, that the BG system is crucially implicated in translating goal-directed behaviours into well-learned responses [77, 78], the possibility that the mesencephalic-BG closed-loops may play a primary role in implicit learning phenomena observed in selective attention has been put forth only recently [50, 79].

**Fig. (1):**
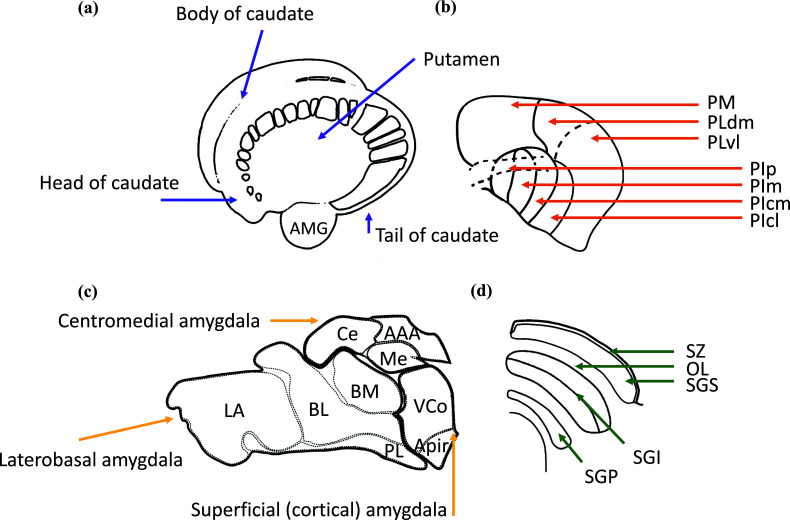
The anatomical subdivision of the primate striatum, pulvinar, amygdala, and superior colliculus. (**a**) The striatum is formed by the caudate nucleus and putamen nucleus. The caudate nucleus is, in turn, divisible in the head, body and tail of the caudate; (**b**) The pulvinar complex comprises several subnuclei: the pulvinar inferior centro-lateral, PIcl; pulvinar inferior centro-medial, PIcm; pulvinar inferior medial, PIm; pulvinar inferior posterior, PIp; pulvinar lateral dorso-medial, PLdm; pulvinar lateral ventro-lateral, PLvl; pulvinar medial, PM; (**c**) the cytoarchitecture of the amygdala includes several subnuclei: the lateral nucleus, (La); the basolateral nucleus (BL); the basomedial nucleus (BM); The paralaminar nucleus (PL); the ventral cortical nucleus, (VCo); the amygdalopiriform transition area (APir); the central nucleus, (Ce); the anterior amygdaloid area, (AAA); the medial nucleus, (Me); (**d**) The superior colliculus is composed of six layers: the stratum sonale (SZ); the stratum griseum superficiale (SGS); stratum opticum (SO); stratum griseum intermediate (SGI); and stratum griseum profundum (SGP). Not indicated in the figure, but part of SC also involves the stratum album intermediate (SAI) and the stratum album profundum (SAP).

**Fig. (2):**
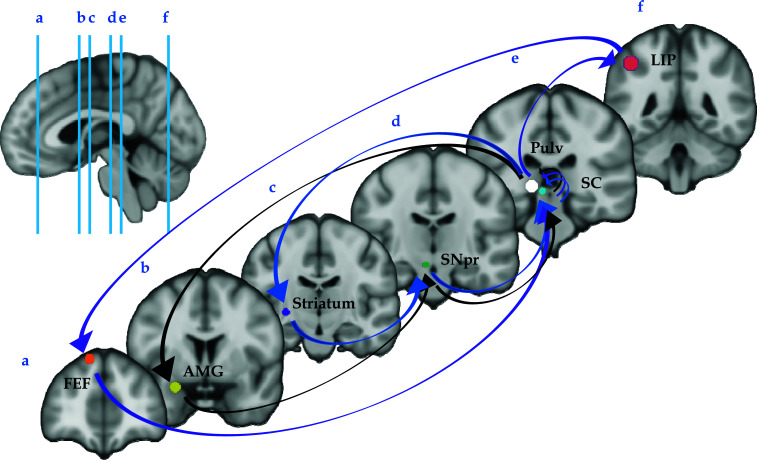
The main collicular human networks involved in selective attention. Through the pulvinar, collicular signals can reach the frontal eye fields (purple arrows), the amygdala (black arrows), and the striatum (blue arrows). All these brain areas, in turn, send descending projections to the superior colliculus, modulating its activity and regulating eye movements and shifts of attention. AMG = amygdala; FEF = frontal eye fields; LIP = lateral intraparietal area; Pulv = pulvinar; SC = superior colliculus; SNpr = substantia nigra pars reticulata.

**Fig. (3):**
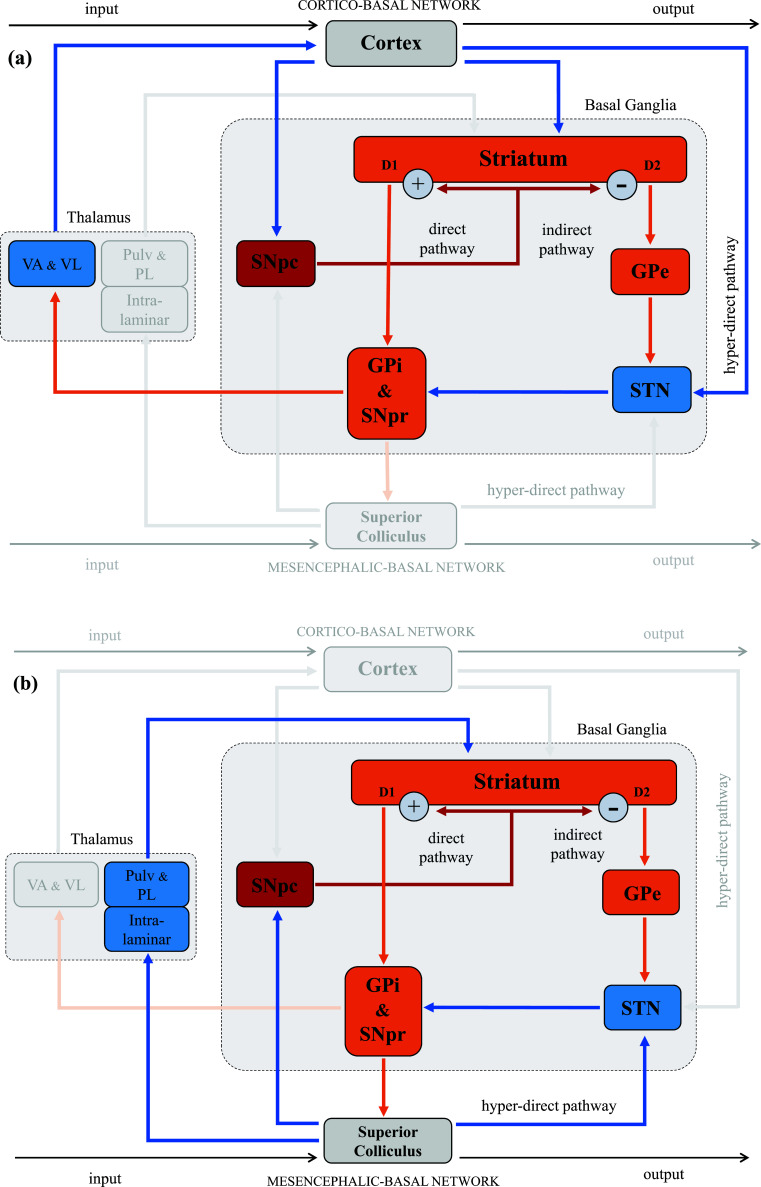
The basal ganglia architecture. The neuronal signal is transmitted from the striatum to the output nuclei of BG through the direct and indirect pathways. In the direct pathway, the striatum sends axons to the GPi and the SNpr, whereas in the indirect pathway, the signal, before targeting the GPi and the SNpr, passes first through the GPe and then through the STN. All striatal territories project to both the GPi and the GPe, while also receiving axons from the SNpc. Finally, in the hyper-direct pathway, signals from the cortex or the tectum can directly recruit the STN, thus bypassing the striatum. (**a**) The cortico-basal architecture; (**b**) The mesencephalic-basal architecture. The glutamatergic structures and projections are indicated in blue. The GABAergic structures and projections are shown in orange; and the dopaminergic structures and projections are demonstrated in red; GPe = globus pallidus externus; GPi = globus pallidus internus; SNpc = substantia nigra pars compacta; SNpr = substantia nigra pars reticulata; STN = subthalamic nucleus; Pulv = Pulvinar; PL = posterior-lateral nucleus; VA = ventral-anterior nucleus; VL = ventral-lateral nucleus.

**Fig. (4):**
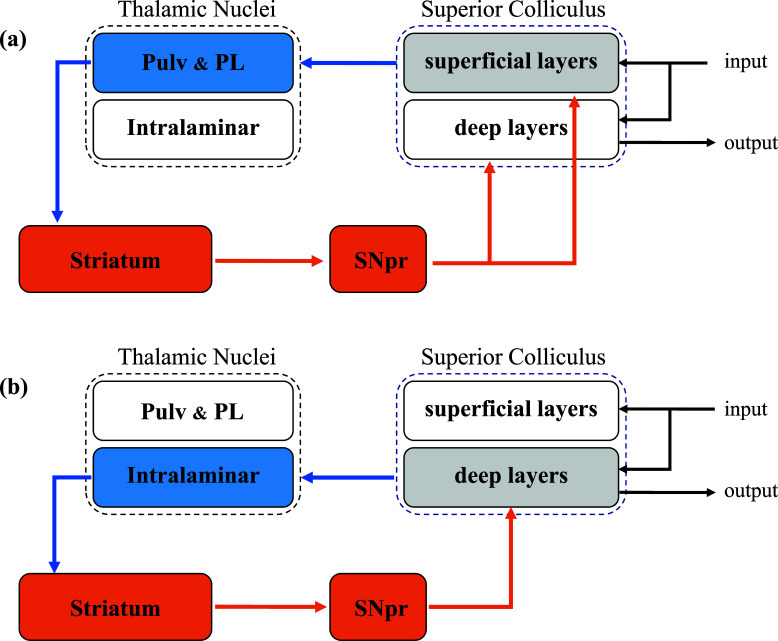
The two main closed loops of the mesencephalic-BG system. (**a**) The closed loop originates from the superficial layers of the superior colliculus. (**b**) The closed loop originates from the deep layers of the superior colliculus. The glutamatergic structures and projections are indicated in blue; and the GABAergic structures and projections are shown in orange; Pulv = Pulvinar; PL = posterior lateral nucleus; SNpr = substantia nigra pars reticulata.

Several recent studies on non-human primates have investigated as to which specific striatal territories concur in modulating SC activity during the selection of visual stimuli, highlighting two main configurations of striatal neurons (Fig. **1**): a first group located in the head of the caudate and involved in the selection of stimuli based on flexible (short-term) abstract rules, and a second group instead located in the posterior putamen and the tail of the caudate and involved in the selection of stimuli based on their stable (long-term) values acquired through repeated individual experience [7, 80, 81]. The head of the caudate seems to play a crucial role in modulating SC activity in situations where the relevance of stimuli changes frequently, thus requiring strategy switching [81, 82]. In fact, chemical and electrical inactivation of this striatal portion leads to a loss in selecting visual stimuli based on reward-related short-term information, sparing the selection of stimuli whose reward-related value has already been consolidated. On the contrary, inactivating the tail of the caudate results in opposite effects, suggesting that this subpopulation of striatal neurons is instead crucially involved in regulating shifts of attention towards stimuli that are historically relevant [81, 83].

In non-human primates, neurons that process stable (long-term) value of visual stimuli acquired through individual experience were found in the caudate tail, as well as in the posterior portions of the putamen (Put), globus pallidus externus (GPe), SNpr, and SNpc [84-88]. The implicitly learned relevance of stimuli, therefore, seems to be encoded along all the intrinsic circuits of the BG that receive visual input from the superficial (visuosensory) layers of the SC Notably, these posterior circuits mainly contain sensorimotor neurons [61, 62], as reported above. The head of the caudate instead receives collicular input only from the deep layers and is highly interconnected with several cortical areas involved in selective attention, such as the dlPFC, FEF and LIP. Therefore, it has been proposed that one possible role of the mesencephalic-BG closed-loops in selective visuospatial attention may be to extract regularity to create sensory-motor memories [50, 89]. Importantly, neurons involved in processing stable (long-term) values of stimuli acquired through experience and capable of triggering automatic shifts of attention towards previously rewarded visual stimuli have been recently found also in the human striatum [90], thus suggesting a neural mechanism conserved across species. The evolutionary value of this mechanism lies in the possibility of making this cortical process automatic and flexible. Once sensorimotor memories are formed and settled in the sensorimotor mesencephalic-BG closed-loops, early visual input from the superficial layers of SC can recruit a rapid automatic modulation of the output of the priority maps located within the deep layers of SC, thus reducing cognitive load without losing the possibility of responding in an adaptive way [50].

We implicitly learn from experience that in certain situations, some stimuli may be relevant, whereas others tend not to be, albeit salient from an evolutionary standpoint [73, 91, 92]. This implicitly acquired automatic selection of visual stimuli seems to be managed by the sensorimotor mesencephalic-BG closed-loops [50, 80].

## 5 THE CONTRIBUTION OF THE AMYGDALA TO SELECTIVE ATTENTION: THE AUTOMATIC CODING OF CONTEXTUAL CUES.

The Amg prioritizes processing emotional or salient signals from the environment through reentrant projections to sensory cortices [93-96]. An Amg response can be triggered through a “double way” of information processing: a fast “low road” from the thalamus to the Amg and a slow “high road” from the thalamus to the neocortex and then to the Amg [97-100]. The low road is the one through which the signal is processed faster, but also in less detail. It is a pathway that allows us to activate our bodies quickly in order to respond promptly to a threat. The high road is slower, but also more precise and systematic, allowing for a cautious and thoughtful assessment of the potentially adverse stimulus [97-100].

As introduced before, the SC, Pulv, and Amg have been identified as nodes of a primate subcortical route to the Amg that bypasses the cortex and participates in the fast and coarse elaboration of evolutionary emotional stimuli [96, 101]. They consistently coactivate in healthy adults [101-104], as well as in cortically blind patients [105-107], when presented with emotional stimuli, such as angry or fearful faces [108]. Further, investigations on sensory unawareness have shown consistently that unseen emotional stimuli elicit activity in the Amg, often along with activity in the SC and Pulv [93, 101, 109-121].

**Fig. (5):**
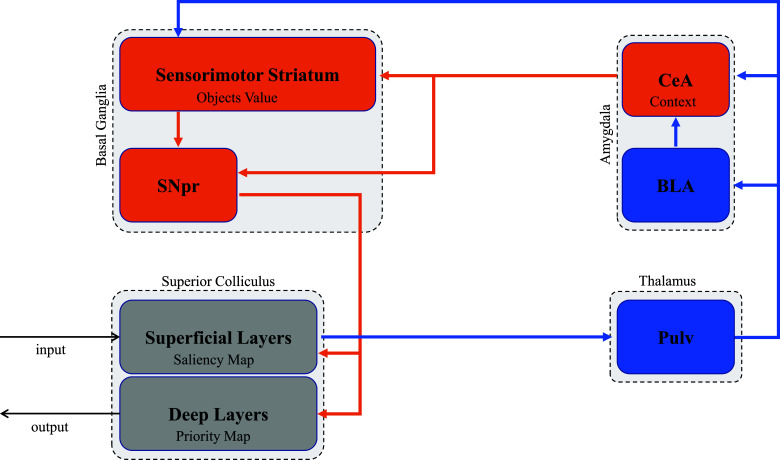
The role of the amygdala in implicit selective attention. Early visual input from the superficial layers of the superior colliculus can recruit the activity of the neurons in the centromedial amygdala that encode the contextual clues based on the specific valence acquired through individual experience. At the same time, early collicular input can also recruit the sensorimotor memories stored in the sensorimotor striatum, which instead encodes the object's values. The two pieces of information are probably integrated within the substantia nigra pars reticolata, which can, therefore (automatically) modulate the priority map output in the superior colliculus, taking into account both the specific object values and the context in which they are present. The glutamatergic structures and projections are shown in blue, and the GABAergic structures and projections are indicated in orange. BLA = Basolateral nucleus of amygdala; CeA = Centromedial amygdala; Pulv = Pulvinar; SNpr = substantia nigra pars reticulata.

Studies on animals have demonstrated that both the superficial and deep layers of SC are connected predominantly to the inferior and anterior Pulv [122-125], with the inferior Pulv receiving fibers and re-projecting to the basolateral (BLA) nucleus of the Amg [45, 108, 126-133]. For the Pulv-Amg pathway, the greatest number of fibers terminate in the inferior Pulv and in the left BLA or right centromedial amygdala (CeA) [108]. Moreover, Amg sends axons to the BG, including the tail of the caudate, the GPe and the SNpr [134-138]. Through these projections (Fig. **5**), the Amg can modulate SC; in fact, in monkeys, chemical inhibition of CeA neurons suppresses saccadic eye movements, whereas optical stimulation of the CeA neurons or of the axon terminals in the SNpr facilitates them [139].

Recent evidence suggests that a primary role of the primate Amg is to modulate selective attention by processing emotional cues [139, 140]. Maeda and colleagues found within the Amg of non-human primates, especially in the CeA, neurons that are activated differentially by the emotional context, based on the specific valence acquired through experience. They found neurons selectively sensitive to the dangerous-safe dimension, selectively sensitive to the rich-poor dimension, and sensitive to both dimensions. Importantly, the activity of these neurons occurred early (about 100 ms after the scene appearance) and it was negatively correlated with the reaction time of shifts of attention towards stimuli previously rewarded [140]. Due to the anatomical connectivity between Amg and BG, information on object value and context is probably integrated at the level of the mesencephalic-BG network output [139, 140]. In other words, the Amg, mostly the CeA, contributes to attentional selection by encoding whether a specific context is potentially dangerous or safe, rich or poor, based on previous individual experiences [140]. As a matter of fact, signals from the Amg are integrated with the BG outputs, probably in the SNpr [139], thus allowing an automatic selection of the stimuli that takes into account both the specific values of objects and the context in which they are present.

## 6 IS AWARENESS NECESSARY FOR THE FORMATION OF IMPLICIT SENSORY-MOTOR MEMORIES?

The above data show that one of the main roles of the BG system in selective attention is to automate the resource-dependent attentional processing managed by the cortex and to form sensorimotor memories that are capable of fast modulating the priority map output in the deep layers of the SC [50]. An open question is whether awareness is necessary for the formation and/or retrieval of these memories. In this regard, clues can be gained from some clinical conditions, such as blindsight (BS), unilateral spatial neglect (USN) and anosognosia for hemiplegia (AHP).

These clinical populations seem to suggest that alterations in the attentional networks support the dysfunction of the metacognitive-executive system but still allow the formation of implicit memories [141]. Importantly for our purposes, there is a strong association between lack of or reduced awareness and brain lesions involving cortical and subcortical structures [142, 143]. Considering that lesions in these areas are often associated with disturbances related to visuospatial processing, online monitoring of information, and retrieval of bodily and autobiographical memories, the role of BG and SC appears consistent with the wide variability of symptoms observed in anosognosia or abnormalities in conscious information processing [144].

### 6.1 The Blindsight Phenomenon

BS is a clinical condition in which patients with a lesion to the primary visual cortex (V1) manifest implicit (*i.e*., without subjective awareness) residual visual abilities [145-147]. Specifically, they retain sensitivity within their visual field, including recognition and spatial localization of stimuli, pointing, grasping, discrimination of orientation, shape, form and wavelength, encoding of direction and speed of movement, obstacle avoidance, and discrimination of facial and bodily expressions [106, 146, 148-151]. The direct pathway from SC to the inferior Pulv appears to be involved, at least in part, in mediating those residual visual abilities in patients with damage to V1 [152]. Indeed, ablation of the SC or reversible inactivation of the connection between SC and the Pulv impairs residual vision after a V1 lesion [153, 154] and leads to impaired saccades or target attainment in the blind field of monkeys with V1 lesions [153]. Thus, stimuli in the blind field may recruit spatial attention in the early phase of visual information processing. This suggests that the appearance of secondary reinforcing visual cues could intervene in the acquisition of novel instrumental behavior. Recently, Kato and colleagues found that the monkeys’ability to discover the location of the target zone was retained when the conditioned stimuli were subsequently presented in the lesion-affected visual field [154]. This finding strongly suggests that early visual input from the superficial layers of SC can still recruit sensorimotor memories stored in the striatum, despite cortical damage and lack of visual awareness.

### 6.2 The Unilateral Spatial Neglect

The USN is a neurological disorder in which, as a result of brain damage mainly in the right parietal lobe, patients show deficits in spatial attention orientation and spatial representations in the contralesional visual hemifield. Despite their inability to consciously detect the contralesional stimuli, patients with USN can still process and respond to perceptual and semantic features of the neglected stimuli without being aware of it [155-158]. Notably, contralesional stimuli that are perceptually or biologically salient may overcome inattention symptoms [159-163], as well as previously fear-conditioned stimuli [164]. Therefore, the early (rapid) sensory processing managed by the SC-Pulv-Amg route still occurs. Further, a similar effect has also been observed with rewarded stimuli [165], and USN patients would appear to be as sensitive as healthy individuals to the distribution of targets even in the neglected field, responding more quickly when targets appear in the most likely region than when targets appear in the least likely region [166]. This last phenomenon of optimization of visual processes is achieved by contextual cueing, which interplays selective attention and implicit learning [167]. As a robust memory for visual context that exists to guide spatial attention, contextual cueing has been shown to direct spatial attention towards embedded targets when there is a high degree of regularity between targets and distractor context in visual search tasks [168, 169]. Rather than being conscious or intentional, this contextual knowledge is acquired implicitly [8]. The observed facilitation may occur during perceptual encoding of the input as a result of contextual cueing that automatically redirects the saccadic eye movements necessary for target discrimination [166]. In other words, it could be managed locally by the sensorimotor mesencephalic-BG closed-loops that originate from the superficial layers of SC and by the CeA neurons that encode contextual cues [139, 140], as argued above.

### 6.3 Anosognosia for Hemiplegia

AHP is a neurological condition in which patients neither perceive nor record their paralysis. However, despite their lack of awareness, they often adjust their behavioral performance over time unknowingly [170], suggesting “*implicit awareness*” of motor impairment. AHP might paradoxically be accompanied by cognitive understanding or representation of signs and deficits, yet not explicitly expressed: in such cases, hemiplegic patients may demonstrate implicit sensory-motor formation by their actions or expressions [170, 171]. For example, Nardone and colleagues (2007) tested a group of AHP patients using an attentional-capture paradigm with hemiplegia-associated words as distractors and demonstrated that AHP patients are still prone to the effect of implicit learning of selective attention: patients displayed significantly higher latencies than healthy subjects when target stimuli were presented with emotionally threatening distractors (*i.e*., hemiplegia-associated words), but not when the distractors were emotionally neutral (*i.e*., when they did not refer to the acquired hemiplegia) [171]. Evidence of this interference (increased latency) could be traced back to implicit associations recently learned during daily living.

Those findings suggest that the mesencephalic-BG closed-loops can still operate, frequently prioritizing relevant stimuli and automatically implementing adaptive responses.

## OUTSTANDING QUESTIONS AND INTERIM CONCLUSION

A growing body of evidence implicates specific brain circuits in humans' introspective and conscious experiences of visual stimuli [172]. In response to recurrent sensorimotor patterns of perception and action, emotional and cognitive structures and processes emerge. It is through sensorimotor coupling between organisms and their environments that endogenous dynamic patterns of neural activity are formed and that ever-new sensorimotor memories are formed.

The role of the BG in forming sensorimotor memories is increasingly being demonstrated as a primary feature of this system since one of its core functions is to extract regularity from the external environment [50]. This neural mechanism appears to be ancient; in fact, the effects of implicit learning on selective attention have been observed in birds, amphibians, reptiles, and fish [79], other than in mammals [7], and the neural structures sustaining this mechanism are evolutionary and shared among all species. Learning to prioritise frequently relevant stimuli, ignoring those that, although originally salient, become irrelevant, and using environmental cues to predict whether a given situation could be potentially dangerous or rich, are all behaviours that can significantly increase the survival odds. The evolutionary advantage of the mesencephalic-BG system could therefore be traced to the possibility of automatizing these functions, making the selection of relevant stimuli and the implementation of related responses faster. Therefore, evolution seems to have exploited a subcortical-cortical mechanism to automate even attention-related fronto-parietal activity.

A subcortical mechanism that detects potentially environmentally threatening stimuli by using the collicular-pulvinar-amygdala network [173] is likely to serve as a mediator for covert attentional orientation [174] and automatic raw processing of value-based stimuli [43, 107], ensuring survival and adaptation to the external environment. The mesencephalic-BG system contributes to selection processes by automatizing the top-down modulation of priority map outputs in the deep layers of SC through the formation of sensorimotor memories, which, once formed and stored in the sensorimotor striatum, can be automatically recruited by early input from the SC [50]. This advantage also seems maintained in clinical populations characterised by focal cortical lesions and preserved subcortical areas and manifesting a lack of awareness of specific behavioural responses, such as in the AHP. In such a case, implicit awareness of motor dysfunctions could originate from a dissociation between attentional, executive and mnemonic components [175]. Studies on AHP or BS and USN, which are not an all-or-nothing phenomenon but have partial and fluctuating trigger-tie responses, demonstrate implicit learning and consequent manifest behaviours [175]. Attention, therefore, has been paid to the relationship between anosognosia and hemiplegia [176-178], anosognosia and neglect [179-181], and anosognosia and blindsight [182]. These disorders may occur simultaneously, and a partially common underlying process has been hypothesised [144].

As a result of several studies analyzing reduced awareness of sensory, motor, and cognitive impairments, it has become increasingly apparent that abnormal conscious processing of information can be caused by disruptions of several cognitive mechanisms and anatomical networks [144]. The three conditions may all be affected, though in different ways, by inadequate neuromodulation between the SC, BG, subthalamic nuclei, and higher-order cortices and may paradoxically maintain signs of coarse understanding or representation of simple attention-guided behaviours. This “*emergent awareness*” based on implicit learning allows AHP patients to recognize their deficits when they have been asked to perform an action and realise their errors [183], or they must attempt dangerous actions [184], while influencing the manifest behaviour in patients with USN or BS, given that selective attention and error processing mechanisms are intrinsically automatic [185]. As a result, ecological behaviour depends on access to a plurality of information sources (such as proprioception, visual attention and motor attention), which are simultaneously engaged at explicit and implicit levels. Sensorimotor memories are usually consolidated in the light of *action-* and *self-monitoring* proper functioning. In the case of impairment, varying degrees and types of awareness dysfunction may occur [141, 144, 175]. Those inferences prove to be robust beyond the heterogeneity of the neuropsychological measures used to evaluate implicit sensory-motor memories or awareness and the variation in the amount of information available in the three pathological models (for example, USN has a much longer tradition of implicit memory evaluation than AHP). The neural underpinnings of implicit sensory-motor memory and implicit (emergent) awareness remain to be clearly disambiguated. Therefore, new functional neuroimaging studies involving implicit learning tasks that underline the role of selective attention in subjects with different levels of impaired awareness (or abnormal conscious processing of information) are needed.

There is still a question as to how explicit and implicit levels are integrated. Of particular interest will be studies on how mesencephalic-BG closed-loops intervene and support other subcortical-cortical networks to allow the aware subjective experience [186]. In particular, new research is needed to explore the association between implicit sensory-motor memory impairment and the disruption of brain regions involved in selective attention and awareness. The clinical implications of this future research are considerable. Reduced awareness leads to high noncompliance rates during the first 4 years after diagnosis [187] and poor prognosis and rehabilitation [188]. By contrast, the proper mesencephalic-BG closed-loop function could facilitate sensory-motor memories and, consequently, treatment options, both acute and post-acute, pharmaceutical and non-pharmaceutical. By mobilizing the dopaminergic system, BG supports the reinforcement of positive outcomes, which promotes a success-driven learning system that limits decay after learning [189]. As a result of the dopaminergic input from the substantia nigra and spatial information through the cortico-striatal connections, reward expectations modulate striatal projection neurons' activity [190]. This modulates the inhibitory output of the BG, which directs attention toward rewarded items [190]. Considering that dopamine accumulates gradually and lasts for long periods [191], it may facilitate the formation of long-term sensory-motor memories that contribute to proper behaviors in daily living. Finally, implicit (emergent) awareness may facilitate rehabilitation and recovery as patients may exhibit an inclination to take part in the therapeutic process to tackle neurological dysfunctions because of the well-functioning of circuits we have described.

## LIST OF ABBREVIATIONS

Amg: Amygdala
BG: Basal Ganglia
FEF: Frontal Eye Fields
GPe: Globus Pallidus Externus
IPS: Inferior Parietal Sulcus
Pulv: Pulvinar
SC: Superior Colliculus
SNpc: Substantia Nigra Pars Compacta
SNpr: Substantia Nigra Pars Reticulata
USN: Unilateral Spatial Neglect
